# A Review of Perspectives on the Use of Randomization in Phase II Oncology Trials

**DOI:** 10.1093/jnci/djz126

**Published:** 2019-06-20

**Authors:** Michael J Grayling, Munyaradzi Dimairo, Adrian P Mander, Thomas F Jaki

## Abstract

Historically, phase II oncology trials assessed a treatment’s efficacy by examining its tumor response rate in a single-arm trial. Then, approximately 25 years ago, certain statistical and pharmacological considerations ignited a debate around whether randomized designs should be used instead. Here, based on an extensive literature review, we review the arguments on either side of this debate. In particular, we describe the numerous factors that relate to the reliance of single-arm trials on historical control data and detail the trial scenarios in which there was general agreement on preferential utilization of single-arm or randomized design frameworks, such as the use of single-arm designs when investigating treatments for rare cancers. We then summarize the latest figures on phase II oncology trial design, contrasting current design choices against historical recommendations on best practice. Ultimately, we find several ways in which the design of recently completed phase II trials does not appear to align with said recommendations. For example, despite advice to the contrary, only 66.2% of the assessed trials that employed progression-free survival as a primary or coprimary outcome used a randomized comparative design. In addition, we identify that just 28.2% of the considered randomized comparative trials came to a positive conclusion as opposed to 72.7% of the single-arm trials. We conclude by describing a selection of important issues influencing contemporary design, framing this discourse in light of current trends in phase II, such as the increased use of biomarkers and recent interest in novel adaptive designs.

The classical paradigm of oncological drug development comprises three phases of clinical trials. In phase I, a treatment’s toxic effects are assessed and the recommended dose(s) for subsequent trials determined. Phase II provides the first assessment of a regimen’s efficacy, with success typically resulting in a confirmatory phase III trial being conducted. Phase II trials thus play a pivotal role, providing the principal evidence for deciding whether to carry out a large phase III trial. The consequences of an incorrect decision at this time can be far-reaching: halting the development of an effective regimen could deprive future patients of a valuable treatment option, whereas continuing to develop a futile regimen could waste substantial resources. Accordingly, the optimal approach to conducting phase II trials has received much attention.

Historically, given the scarcity of effective anticancer agents in the 1950s, the accepted perspective was that phase II should act as a screening process to efficiently weed out inactive regimens (1,2). For this, a short-term surrogate endpoint for long-term clinical benefit was required. A binary tumor response indicator, later formally defined via RECIST (3), became the endpoint of choice (2). Furthermore, Gehan offered in 1961 a design that would long remain the primary approach to phase II oncology trial design (1). Based on the belief of the day that a response rate of less than 20% was not promising, he recommended that 14 patients be recruited and observed for tumor response. If no response was observed, this would ascertain with 95% confidence that the true response rate was less than 20%. With at least one response observed, a second stage of recruitment and observation would be conducted to accurately estimate the true response rate.

Unfortunately, Gehan’s design provided no guidance on whether the observed response rate was clinically meaningful. Consequently, as the number of available effective oncology drugs increased during the 1970s, phase II trials shifted to demand a higher standard of evidence of potential clinical benefit (2). The prevailing techniques for achieving this were Simon’s two-stage single-arm designs (4), which were constructed to allow a formal hypothesis test to be conducted on the tumor response rate. Simon’s designs, like Gehan’s, experienced a sustained period as the preferred approach in phase II. Thus, unlike in many disease settings, phase II cancer trials were traditionally nonrandomized. Moreover, they almost all used tumor response as their primary outcome (5). By the mid-1990s, however, questions over the optimality of this convention began to arise.

In this article, we focus on these questions, in particular examining opinions on the role of randomization in phase II. To provide a comprehensive overview of this subject, we base our discourse on a completed narrative literature review (see Supplementary Methods and Supplementary Tables 1–5 for details on this review). We proceed by describing one of the primary reasons for the now evident changes in phase II design. We then discuss the arguments put forth for and against the use of randomization, along with the real-world evidence and statistical investigations that complicate this debate. Our discourse then turns to consider the current design of phase II oncology trials and how such design aligns with past opinions on best practice. We conclude by highlighting current issues that are likely to influence changes in the design of future phase II trials.

## Changing Phase II Design

Arguably the principal driver of change in phase II oncology trial design was the advent of molecularly targeted agents (MTAs). Classic anticancer agents were cytotoxic; thus it was considered reasonable to assess their efficacy via their ability to shrink tumors, and the fact that tumors do not typically spontaneously shrink meant many accepted the lack of a randomized comparator arm. However, it was anticipated that the new MTAs may often be cytostatic, delaying tumor progression rather than leading to tumor shrinkage (6,7). Accordingly, it was feared that the use of tumor response as a primary endpoint may lead to many novel regimens that do increase clinical benefit being rejected from further consideration (8–11). Thus, it was contended that a new approach was required in phase II for cytostatic agents.

Initially, the proposed approaches varied greatly, with some authors even suggesting that phase II be skipped (12). Unsurprisingly, such suggestions were not widely favored. It was acknowledged that although this may seem a desirable means of reducing development plan sample sizes, the ever-rising cost of phase III studies, and the scarcity of available patients to enroll in trials, would make it impossible to test all regimens in this manner (9,13,14). Further, given a preliminary efficacy assessment could be acquired in phase II using a relatively small number of patients, it would be unethical to expose large numbers of patients to a regimen for which little evidence of efficacy exists (9).

More reasonably, calls for the use of an alternative primary endpoint in phase II trials of cytostatic agents soon grew (13,15). In particular, progression-free survival (PFS) was much advocated because an assessment of time until disease progression would more accurately capture the benefits of cytostatic agents. Furthermore, PFS, it was argued, would be more efficient to estimate than overall survival and would not be affected by salvage treatment. However, PFS is highly dependent on a disease’s natural history. It was thus contended that with this necessary change in endpoint, so came a necessary change in design to use randomization (11,16–19). With this, one of the primary reasons randomization has been advocated for in phase II was apparent.

## Arguments for and Against the Use of Randomization in Phase II

Along with changes proposed to phase II design because of the rise of MTAs, appeals for the use of randomization no matter the regimen under investigation or the chosen primary endpoint also increased. Here, we discuss the arguments for and against randomization that have appeared in the literature, providing an overview in Table 1. Note that we concentrate our discussion on randomization as a means of formally comparing treatments using a hypothesis test because the majority of articles have focused on the use of randomization for this purpose. Thus, for brevity, when we now refer to randomized designs, we mean randomized comparative designs. However, many of the following points are also relevant to a debate on the utility of randomized noncomparative designs.

**Table 1.** A summary of the key arguments or counterpoints that have historically been put forward for and against the use of randomization in phase II oncology trials

Consideration	For randomization	Against randomization
Molecularly targeted agents may often be cytostatic and not in general lead to tumor shrinkage.	PFS* should be the preferred primary outcome for cytostatic agents, which in turn makes randomization preferable.	The use of tumor response is still appropriate because several cytostatic agents have led to statistically significant tumor regression.
Success in phase II should reliably predict that clinical benefit will be observed in phase III.	The classical single-arm paradigm has performed poorly in predicting clinical benefit in phase III; randomized designs would perform better.	No evidence is available to suggest the use of randomization in phase II has improved success in phase III.
Randomized phase II designs appear more similar to phase III designs than conventional phase II designs.	A highly statistically significant *P* value from a randomized trial would provide a strong case for seeking regulatory approval.	Investigators may incorrectly interpret the results of randomized phase II trials as though they are from a large phase III study.
Single-arm trials use historical control data to specify their design.	Reliance on historical control data for setting target response makes results of single-arm trials unreliable.	For several diseases, well-established resources allow for determination of historical response to treatment.
Prognostic factors can differ substantially between patients, and these are often strong predictors of response to treatment.	Randomization should balance prognostic factors between arms, allowing for more reliable assessment of efficacy. Attempts to account for such covariates in single-arm data using modeling are unreliable.	Randomization cannot guarantee prognostic factors will be balanced, especially in smaller trials, and modeling can be used to account for such variables in single-arm studies.
There is a trade-off between trial complexity and quality.	In conducting a randomized trial, you “get what you pay for,” with better quality data accrued.	Single-arm trials are simpler and easier to conduct.
Clinical trials should be conducted with as few participants as required to control type-I/II error rates to specified levels.	One-sided testing with increased type-I/II error rates allows randomized trials to be conducted with achievable sample sizes. Only in a randomized trial are these error rates ever known.	Single-arm trials require a much smaller sample size, and modifying error rates to reduce that required by a randomized trial would only increase failure in phase III.
Single-arm designs should be preferred from an ethical standpoint.	In general, there is equipoise, making randomized trials entirely appropriate.	Randomized trials are not ethical when large responses have been observed previously because not all participants have access to a potentially better treatment.

* PFS = progression-free survival.

At the center of most discussions on phase II design has been the key assumption of a single-arm trial: The historical controls used to design the current study provide a fair representation of the probable outcome for patients in the new trial if they were given the same control treatment. Many have noted that, because of improvements in the standard of care, earlier detection rates, institutional variabilities, and differences in prognostic factors, only in rare scenarios would historical controls provide such a representation (20–23).

Potential differences in prognostic factors between historical controls and concurrent participants were seen as particularly problematic because it was well recognized that unknown prognostic factors can profoundly affect a single-arm trial’s error rates (23,24). A modeling-based approach was suggested as a possible solution to accounting for such prognostic variables (25). However, although proponents of randomization acknowledged that this could improve on simple unadjusted comparisons to historical controls, given the majority of interindividual variability remains unexplained after accounting for all measured predictors (8,16,26,27), they argued that such adjustment procedures were not widely applicable or reliable (20,26). So too did others note that the desire for a new agent to “look good” could manifest itself in setting the historical response rate too low (28,29) or the enrollment only of patients who “look promising” (30). Each of these problems, it was maintained, would not be an issue in a randomized trial (17,18,20,22–24,26,30–33).

Nonetheless, numerous publications still argued in favor of single-arm designs (23,34–37), claiming that they can be readily used given high enough confidence in the historical data (23, 34). A melanoma overall survival database was in particular a heralded means through which successful single-arm studies had been conducted (25). In addition, those in favor of single-arm designs noted flaws in the supposed advantages of randomization, arguing that the purported ability of randomized phase II trials to balance prognostic factors was an illusion, because the majority of such trials used simple randomization, which would be unlikely to provide balance for typical phase II sample sizes (23,36,38–40). Moreover, as was feared about single-arm trials, randomized phase II trials could also feature highly selected patients (17) and easily fall foul of differential loss to follow-up or patient drop-out (41). As a counterpoint, it was argued that randomization was a better way to handle many sources of bias and actually the only way to make detection and quantification of imbalances possible (23).

Randomized phase II trials have also been viewed as troubling because of the possibility that investigators will mistakenly treat their results as though they are from a phase III trial (42), a claim arguably supported by the large number of comparisons performed at the end of noncomparative trials (38). This though, others claimed, should not be an argument against randomization, and in fact a highly statistically significant *P* value from a randomized phase II trial could provide enough evidence for a licensing claim, which is not in general possible from a single-arm study (23).

Perhaps the most common argument against randomizing was its associated cost and complexity, with many fearful of the resultant requisite sample size, the fact that randomized trials can be more complex for patients to understand, and that it may be difficult to find patients willing to be randomly assigned (23,34,35). This viewpoint was supported by a review that found 27% of conducted randomized phase II studies had encountered “sizeable problems” (23). Those in favor of randomization, however, argued that in general randomization would provide more reliable data (8), and because “you get what you pay for,” (33) the increased cost should be accepted (13). Furthermore, this argument ignored the fact that randomizing in phase II could decrease the cost of a project overall (20,26,43) and that whereas it may seem appealing to use single-arm trials to develop drugs quickly, or to allow expedient publication, in the long term we all had more to gain from well-designed, randomized phase II trials (33).

Advocates of randomization further attacked the issue of larger requisite sample sizes by arguing for one-sided hypothesis tests and allowance for increased type-I error rates (9,14,16,17,20,29). Given the purpose of phase II is to screen and select, this was claimed to be entirely appropriate (16) and also logical for ethical reasons (17). As a counterpoint, it was noted that the advantages of increasing error rates to reduce sample sizes in phase II are negated by the cost associated with conducting more phase III trials with inactive agents. However, pro-randomization researchers contended that some control is better than none and highlighted that the type-I error rate in single-arm trials is actually unknown (23,26).

Arguments in favor of single-arm designs were also made on ethical grounds, with some desiring to provide all enrolled patients the experimental regimen (34). Others, though, stated that randomizing may be more ethical because the experimental treatment could be inferior (26), contending that in most cases there is equipoise (33).

Several authors were critical of the approach randomization implies about our method of identifying new treatments. Most prominently, Stewart and Kurzrock argued that using randomized designs in phase II to balance for characteristics that might confound efficacy assessment is the antithesis of personalized medicine and that unless the target required for drug activity is very common or we know for each patient whether his or her tumor expressed the required target, randomization could produce misleading results (44). This argument, though, was not logical to all, with it noted that any experienced group of investigators would identify a subgroup of patients with exceptional outcomes within a randomized trial (33).

Finally, it is important to note that deliberations on what should become the typical primary endpoint in a phase II trial have long interweaved the debate around randomization, a consequence of a desire for improved endpoints on which to select treatments (10,45–50). Here, though, we have limited our discussions to our primary interest: the suitability of single-arm designs in phase II. For an in-depth history of endpoints that could be utilized in oncology trials, we refer the reader to Wilson et al. (51).

## Practical Evidence on the Outcome of Utilizing Randomized or Nonrandomized Designs

Along with the discussions outlined above, several authors have provided evidence on the result of using different design frameworks in phase II in practice. In particular, much evidence has suggested that the classical approach has contributed to the failure of phase II trials to predict long-term survival benefits. Kola and Landis (52) highlighted that 60% of regimens that had “promising” activity in single-arm phase II trials failed to demonstrate superiority in phase III. Zia et al. (53) identified that response rates were regularly lower in phase III trials than in their preceding phase II studies. Maitland et al. (54) analyzed all phase II combination therapy trials published in 2001–2002 and found that despite 72% being deemed successful, the likelihood that an ensuing trial would demonstrate important effects within 5 years was only 3.8%. Ratain (55) pointed to the extremely low positive predictive value of classical II trials. Finally, Vickers et al. (29) identified that 46% of surveyed phase II studies that required historical data did not cite the source of these data. Even more troubling was their finding that those trials that failed to cite prior data suitably were more likely to declare an agent to be active. For many, this was evidence that a higher bar now needed to be set for progressing to phase III. The solution to attaining this higher bar, it was argued, was randomization in phase II (13,17,20,30,33,56).

Complicating the picture, though, were the many results that favored the use of single-arm designs and tumor response. Goffin et al. (57) identified that higher overall response rates in phase I and II were predictive of regulatory approval in a range of cancer types. Moreover, El-Maraghi and Eisenhauer (50) established, in 89 surveyed phase II trials, higher response rates were predictive of regulatory approval for MTAs. Tsimberidou et al. (58) noted that of 31 drugs approved in 1973–2006 without a randomized trial, 30 remained fully approved. Finally, Chan et al. (59), in examining all phase III trials of targeted therapies against advanced cancers from 1985–2005 and their preceding phase II studies, found that randomization in phase II was not more likely to lead to a positive phase III result.

Consequently, it was argued that success issues in phase III were not due to the use of single-arm trials in phase II but to how these trials were planned and interpreted, and thus we should focus on conducting high-quality single-arm trials (23). Not all were convinced by these presentations, however (20,60), with Gan et al. (23) in particular noting that such reviews are biased because many trials are never published, and it is likely that a higher proportion of randomized phase II trials would be published because of their greater intrinsic value.

## Statistical Investigations of Randomized and Nonrandomized Design Operating Characteristics

Thus, arguments for and against classical phase II designs could readily be made based on the available evidence from design use in practice. Hoping to clarify purely statistical recommendations, several publications have provided quantitative figures on optimal phase II design.

Firstly, Mariani and Marubini noted that the error rates of single-arm but not randomized trials are highly sensitive to the historical control rate assumptions (61). Baey and Le Deley conducted a similar analysis and argued in favor of randomized designs (62). Providing a less favorable view of randomization, Sharma et al. (63) resampled data from a positive and a negative phase III trial. They considered a variety of endpoints and designs, finding that randomized designs performed better for PFS at predicting a positive result for the positive trial but that they had a higher false-negative rate for the negative trial.

Taylor et al. (64) compared single-arm and randomized designs using a simulation study. They found that a randomized design of equal size to a single-arm trial made the correct decision more often and that increasing the sample size was more beneficial to randomized than to single-arm trials. They concluded that in the presence of uncertainty around the historical response rate, and when a larger sample size is possible, a randomized design should be used. Similarly, Tang et al. (65) simulated single-arm and randomized phase II trials using data from a colorectal cancer study. They found that the false positive rate of single-arm trials was two to four times higher under modest drift of several considered patient-selection factors but that randomized phase II trials came at a cost of two to four times the sample size. They also argued that given enough patients, a randomized design was preferable. Sambucini later came to similar conclusions, having taken a Bayesian approach to design comparison (66). However, Pond and Abbasi (67), using a simulation study that modeled numerous potential sources of variability, argued that single-arm and randomized designs were both warranted in certain circumstances.

## Consensus Opinions on Phase II Design

Thus, in spite of much debate, no consensus on the optimal design of phase II trials emerged. Nonetheless, several areas of general agreement are apparent, being for the most part described in two articles released following committee discussions (68,69) and in a table provided by Gan et al. (23). In particular, it was widely accepted that single-arm as well as randomized designs have a part to play in phase II (23,37,69–71), with it typically agreed that the number of randomized phase II trials should increase and PFS should become the conventional endpoint of choice (37).

Most authors believed that single-arm trials were acceptable for single, especially novel, agents when tumor response was expected (17,19,23,69). They would also be useful for rare cancer types, for which it may be challenging to enroll enough patients to conduct a randomized trial (23,34,72), in late disease and salvage settings, and when no standard treatment exists (68,69). Additionally, if data suggested a response rate that was dramatically higher than that for available therapies, we may forego randomization (72). They could also be logical in the admittedly rare situations in which a robust historical database exists (68).

In contrast, randomized designs would be required for combination therapies, because it would otherwise be difficult to distinguish the contribution of the experimental agent from the established component (14,68,73). They would be needed for time-to-event endpoints (3,17,23,69), when there is a lack of available suitable historical data (9,68,72), or when the target population is unclear or heterogeneous (69,74,75). However, investigators were cautioned against conducting small randomized trials, with it propositioned that in general we may need to accept an increase in the size of phase II trials (68).

Finally, many papers agreed that alternative endpoints, outside of tumor response, should be given greater consideration and ultimately be deployed in randomized trials (68,70). Nonetheless, it was also well recognized that although they were promising, many alternative endpoints required additional evaluation and validation before they could be used as primary outcomes in phase II (76), and thus the choice in practice would often remain between tumor response and PFS.

## Figures on the Contemporary Design of Phase II Oncology Trials

The number of available phase II designs is now extensive, with an overview of many provided by Brown et al. (77). Consequently, several articles have sought to examine how frequently particular designs have been used. Together, they provide evidence that arguments for the increased use of randomization have permeated through to practice. Mariani and Marubini found that of 308 phase II trials published in 1997, only four (1.3%) were controlled (5), and Stone et al. examined publications in the *Journal of Clinical Oncology* between 1999 and June 2003 and identified only 23 randomized phase II trials out of approximately 250 studies (16). However, Rubinstein et al. noted in 2011 that 69 (28%) of the then active National Cancer Institute–sponsored phase II trials were randomized (19). In addition, Thezenas et al. (78), Ivanova et al. (79), and Langrand-Escure et al. (80) have all since conducted reviews of phase II trials, each identifying a large proportion that incorporated randomization.

Here, we focus on reanalyzing the data from Langrand-Escure et al. (80), who examined 557 trials published in three top oncology journals from 2010 to 2015. In Table 2 we present an assessment of several design characteristics by design type: single-arm, multi-arm nonrandomized, randomized noncomparative, and randomized comparative studies, which form the main types of design in this dataset. In Table 3 we summarize the choice of primary and coprimary endpoints by the type of treatment under investigation. Our analyses reveal several important figures in relation to the aforementioned opinions on best practice. We provide a summary of these in Box 1.

Box 1. A summary of the key findings, in light of historical recommendations, from the reanalysis of the data from Langrand-Escure et al. (80)

Finding

102 (32.4%) of the single-arm trials were of combination therapies.

47 (66.2%) of the trials employing progression-free survival (PFS) as a primary or coprimary outcome used randomized comparative designs.

18 (5.7%) of the single-arm trials used PFS as a primary or co-primary outcome.

The median number of patients analyzed in the randomized comparative trials was 131 compared with 43 for the single-arm trials.

29 (28.2%) of the randomized comparative trials came to a positive conclusion compared with 152 (72.7%) of the single-arm trials.

More than 50% of the trials of cytotoxic, combination, and targeted therapies used either tumor response, PFS, or a dichotomized version of PFS as a primary or coprimary outcome.

35 (34%) of the trials of targeted therapies used tumor response as a primary or coprimary outcome, whereas 16 (15.5%) used PFS.

**Table 2.** A summary of the characteristics of phase II oncology trials by type of design used, based on a reanalysis of the data from Langrand-Escure et al. (80)*

Characteristic	Single-arm, % (IQR)	Multi-arm nonrandomized, % (IQR)	Randomized noncomparative, % (IQR)	Randomized comparative, % (IQR)	*P*†
Type of therapy					
Cytotoxic	84 (26.7)	13 (26.5)	24 (31.6)	27 (23.1)	.02
Combination therapy	102 (32.4)	11 (22.4)	27 (35.5)	53 (45.3)	
Targeted therapy	62 (19.7)	17 (34.7)	8 (10.5)	16 (13.7)	
Other	67 (21.3)	8 (16.3)	17 (22.4)	21 (17.9)	
(Co-)Primary endpoint‡					
Tumor response	129 (41.0)	18 (36.7)	26 (34.2)	18 (15.4)	—
Dichotomized PFS	23 (7.3)	6 (12.2)	9 (11.8)	5 (4.3)	
PFS	18 (5.7)	1 (2.0)	5 (6.6)	47 (40.2)	
Other	159 (50.5)	26 (53.1)	38 (50.0)	54 (46.2)	
Positive result	152 (72.7)	25 (83.3)	39 (70.9)	29 (28.2)	<.001
Median type-I error rate (IQR)	5 (5–10)	5 (5–10)	7.1 (5–10)	5 (5–10)	.04
Median type-II error rate (IQR)	14 (10–20)	10 (9.8–12.5)	10 (10–20)	20 (15–20)	<.001
Median patients analyzed (IQR)	43 (34–58)	72.5 (49.8–111)	92 (72.8–135)	131 (89–182)	<.001

* Note that the dataset from Langrand-Escure et al. (80) comprises 557 trial reports. IQR = interquartile range; PFS = progression-free survival.

† In addition, *P* values for either a chi-squared test of row-column independence or a Kruskal-Wallis test of stochastic dominance, as appropriate, are reported for each of the characteristics. All statistical tests were two-sided.

‡ The percentages for the (co-)primary characteristic section do not add up to 100% because the categories are not mutually exclusive given the allowance for co-primary endpoints, and consequently a *P* value is omitted for this section.

**Table 3.** A summary of the choices of primary/coprimary endpoints in phase II by the type of treatment under investigation, based on a reanalysis of the data from Langrand-Escure et al. (80)*

(Co-)Primary endpoint	Cytotoxic No. (%)	Combination therapy No. (%)	Targeted therapy No. (%)
Tumor response	69 (46.6)	53 (27.5)	35 (34.0)
Dichotomized PFS	5 (3.4)	18 (9.3)	16 (15.5)
PFS	10 (6.8)	38 (19.7)	16 (15.5)
Other	67 (45.3)	92 (47.7)	44 (42.7)

* The percentages do not add up to 100% because the categories are not mutually exclusive given the allowance for co-primary endpoints. PFS = progression-free survival.

Firstly, against prevailing opinion, a smaller proportion of targeted therapies were tested using randomized comparative designs (13.7%) than cytotoxic therapies (23.1%), and 102 (32.4%) of the single-arm trials were of combination therapies (Table 2). Recommendations on the utilization of randomization with PFS appear to have been better heeded, with 47 (66.2%) of the trials employing PFS as a primary or coprimary outcome being randomized comparative. However, 18 (5.7%) single-arm trials had PFS as a primary or coprimary outcome.

The randomized comparative trials on average analyzed approximately three times as many patients (median = 131, inter-quartile range [IQR] = 89, 182) as single-arm trials (median = 43, IQR = 34, 58), exemplifying an ability in many circumstances to overcome concerns around increased requisite sample sizes (Table 2). Additionally, though there is evidence that different type-I error rates have been used across the four design types (Kruskal-Wallis test, *P *= .04; see Table 2 and Supplementary Figures 1–2), the lower-quartile, median, and upper-quartile values used in the single-arm and randomized comparative trials are equal, suggesting that calls for randomized comparative trials to use increased error rates may not have been realized in the way many authors desired.

Importantly, Table 2 highlights that among the trials that reported a conclusion, the analyzed randomized comparative trials less frequently come to positive conclusions (29 of 103 trials, 28.2%) than single-arm (152 of 209, 72.7%), multi-arm nonrandomized (25 of 30, 82.3%), and randomized noncomparative trials (39 of 55, 70.9%). This, in particular, raises concerns over the choice of historical controls and specification of the hypotheses in the single-arm trials.

Finally, in Table 3 we observe that more than 50% of cytotoxic, combination, and targeted therapy trials have used tumor response, PFS, or a dichotomized version of PFS as a primary or coprimary outcome, with tumor response the most commonly used endpoint for all three treatment types. Also, perhaps surprisingly given previous recommendations, tumor response was used more than twice as often as PFS for targeted therapies.

## Contemporary Factors Influencing Phase II Design

Although the use of randomized designs in phase II has increased, an open question exists as to what this has taught us about randomization’s applicability, particularly given it does not appear to have improved success rates in phase III (81). Furthermore, although there has been much interest in the use of novel endpoints, this has not materialized in the way many hoped it would (82). We may therefore still question whether poor performance in phase III is due to the methods typically used in phase II. Careful design choice in phase II thus remains paramount. In addition, several contemporary articles highlight additional important considerations for future phase II trials.

Firstly, despite historical agreement on the suitability of single-arm designs for rare cancers, whether this should be universally accepted has recently been challenged (83,84). It has been argued that the field of oncology performs a disservice to patients with rare tumors when we accept medications using inferior levels of evidence. It has also been maintained that although randomized trials are difficult to conduct in rare tumors, large randomized trials have been conducted for even ultra-rare tumors, providing reliable results to important questions (83). Thus, randomization has more to do with our expectations than tumor incidence. Accordingly, we should not accept that randomization is not required for rare conditions. Whether randomization can, and will, be more commonly used in rare cancer types remains to be seen.

Moreover, in recent years, there has been increased interest in the use of novel adaptive designs in phase II, such as umbrella and basket designs (85). How this complicates the debate on the use of randomization, and the preferred primary endpoint, remains unclear. Many of the available adaptive designs applicable in phase II are nonrandomized in nature, yet one may expect that adaptation might improve trial efficiency in a way that randomization becomes more feasible. Furthermore, adaptive designs typically require a primary outcome that can be evaluated quickly. This implies that PFS, which has been much argued for historically, may be difficult to use with such designs.

Perhaps most important are considerations around the design of trials that use biomarkers given their evident increased use (80). It has long been argued that randomization would be needed for biomarker-guided trials, not only because historical data would be unavailable but also because of the requirement to validate the biomarker as a predictive marker of efficacy (33,86–88). However, it has been pointed out that conducting randomized trials in prespecified subgroups with very low-frequency biomarkers may be difficult (89) and that randomized assignment may be unnecessary if tumor shrinkage is the primary endpoint, because only predictive factors would be expected to correlate with shrinkage (37).

This debate is further complicated by the emergence of several drugs with extremely high response rates in novel biomarker-guided patient subgroups. Given these remarkable response rates, it has been argued that randomized trials may be unnecessary and unjustifiable on ethical grounds (69,90). However, it has been noted that this remains an extremely unusual situation (33,89,91). Thus, only in rare cases would it be unethical to randomize, and we should not let such findings guide our overall development strategy (33,88), with several authors even advocating for randomized designs to be used more often in phase I (88,92). Nevertheless, Selaru et al. describe a variety of settings in which dramatic activity would be enough to license a monotherapy MTA on the basis of a single-arm trial (93). So too have Simon et al. described the potential future role of tumor response as a basis for licensing (72). They emphasize, though, that the utilization of classical phase II designs remains reliant on the choice of historical controls. Thus, guidelines from regulatory bodies on how to achieve appropriate use of historical comparator groups will be paramount to future trials.

## Discussion

Many years have now passed since the beginnings of the debate on optimal phase II oncology trial design and yet it remains a subject of ongoing deliberation (see, eg, Grossman et al. [94] for a recent examination in the context of glioblastoma). Furthermore, although this debate has contributed to an increase in the number of randomized phase II trials, phase II design for cytotoxic and targeted therapies remains similar, and we remain without evidence that the use of randomized designs in phase II has improved outcomes in phase III.

Little has changed in terms of the importance of resolving this debate. Accordingly, all new guidance on phase II oncology trial design remains extremely valuable. Such guidance will be key to the effectual design of future studies.

## Funding

This work was supported by the Medical Research Council (grant number MC_UU_00002/3 to MJG and APM); the Medical Research Council Network of Hubs for Trials Methodology Research (grant number MR/L004933/1-N97); and the National Institute for Health Research (grant number NIHR-SRF-2015–08-001 to TFJ).

## Notes

Affiliations of authors: MRC Biostatistics Unit, University of Cambridge, Cambridge, UK (MJG, APM); Institute of Health & Society, Newcastle University, Newcastle upon Tyne, UK (MJG); School of Health and Related Research, University of Sheffield, Sheffield, UK (MD); Centre for Trials Research, Cardiff University, Cardiff, UK (APM); Medical and Pharmaceutical Statistics Research Unit, Department of Mathematics and Statistics, Lancaster University, Lancaster, UK (TFJ).

The funders had no role in the design of the study; the collection, analysis, and interpretation of the data; the writing of the manuscript; and the decision to submit the manuscript for publication. The authors have no conflicts of interest to disclose.

The views expressed in this publication are those of the authors and not necessarily those of the National Health Service, the National Institute for Health Research, or the Department of Health and Social Care.

## Supplementary Material

djz126_Supplementary_Data
