# Supplementary material for: A Review of Perspectives on the Use of Randomization in Phase II Oncology Trials
Source: J Natl Cancer Inst. 2019 Jun 20;111(12):1255–62. doi: 10.1093/jnci/djz126 (PMC6910171; doi:10.1093/jnci/djz126)
Supplement: djz126_Supplementary_Data [file djz126_supplementary_data.zip › djz126-Suppl_data/Supplementary_Data.pdf]

Supplementary Methods

Literature review

In order to be able to provide a comprehensive description of the historical and contemporary opinions on the design of phase II oncology trials, and in particular the role randomization should play in such trials, we conducted a thorough literature review. We began with a list of 55 core publications, assembled based on our expert knowledge of the subject area. For these, M.J.G. carried out forward (via Google Scholar) and backward citation checks; examining the Title and Abstract of all identified citations in order to pinpoint additional publications of relevance.

Next, for each of the identified articles of relevance, M.J.G. completed identical forward and backward citation checks, in order to identify further articles. This process was repeated until no extra articles deemed to be of relevance were identified.

In addition, we searched PubMed on 21 February 2018 using the following search term:

> (phase II[Title] OR phase II[Abstract]) AND (single-arm[Title] OR single-arm[Abstract] OR non-randomised[Title] OR non-randomised[Abstract] OR non-randomized[Title] OR non-randomized[Abstract]) AND (randomised[Title] OR randomised[Abstract] OR randomized[Title] OR randomized[Abstract] OR randomisation[Title] OR randomisation[Abstract] OR randomization[Title] OR randomization[Abstract]) AND (oncology[Title] OR oncology[Abstract] OR cancer[Title] OR cancer[Abstract])

The Titles and Abstracts of each of the resultant records were reviewed by M.J.G. in an equivalent manner to the approach described above, with a series of forward and backward citation checks again carried out when relevant.

Ultimately, the above search strategy produced a large number of relevant articles. Given the historical interest in optimizing phase II oncology trial design, the precise number was too large for all to be cited in the article. The full-text of each was read in full by M.J.G. and the key points from each

summarized and categorized. Following initial determination of overall article structure and initial specification of key points to include in each section, several rounds of manuscript refinement determined the final list of records that are cited in the main article.

Access to a Mendeley folder containing details on all identified relevant articles is available from the corresponding author upon request. In addition, the Supplementary Tables list the 55 core articles, the articles extracted from the PubMed search, the cited articles identified via backward citations, and all additional articles that were cited that were not identified as part of our search strategy. Furthermore, they contain all reviewed forward citations, with the exception of those for [1-4], which have together been cited over 9000 times. Given the time-consuming nature of extracting citations from Google Scholar, these forward citations were only reviewed online, and not exported to the Supplementary Tables.

References

[1] Gehan EA. The determination of the number of patients required in a preliminary and a follow-up trial of a new chemotherapeutic agent. *J Chron Dis*. 1961;13(4):346–353.

[2] Simon R. Optimal two-stage designs for phase II clinical trials. *Control Clin Trials*. 1989;10(1):1–10.

[3] Kola I, Landis J. Can the pharmaceutical industry reduce attrition rates? *Nat Rev Drug Discov*. 2004;3(8):711–715.

[4] Wahl RL, Jacene H, Kasamon Y, et al. From RECIST to PERCIST: Evolving considerations for PET response criteria in solid tumors. *J Nucl Med*. 2009;50(Suppl 1):122S–150S.

Supplementary Tables

The Supplementary Tables are available as a separate download.

Supplementary Table 1. 55 core records that the initial forward/backward citation searches were conducted for

Supplementary Table 2. Extracted forward citations for cited records

Supplementary Table 3. Cited records that were identified through backward citation checks

Supplementary Table 4. Additional records that were cited, which were not identified by any other method

Supplementary Table 5. Records identified via the described PubMed search

Supplementary Figures

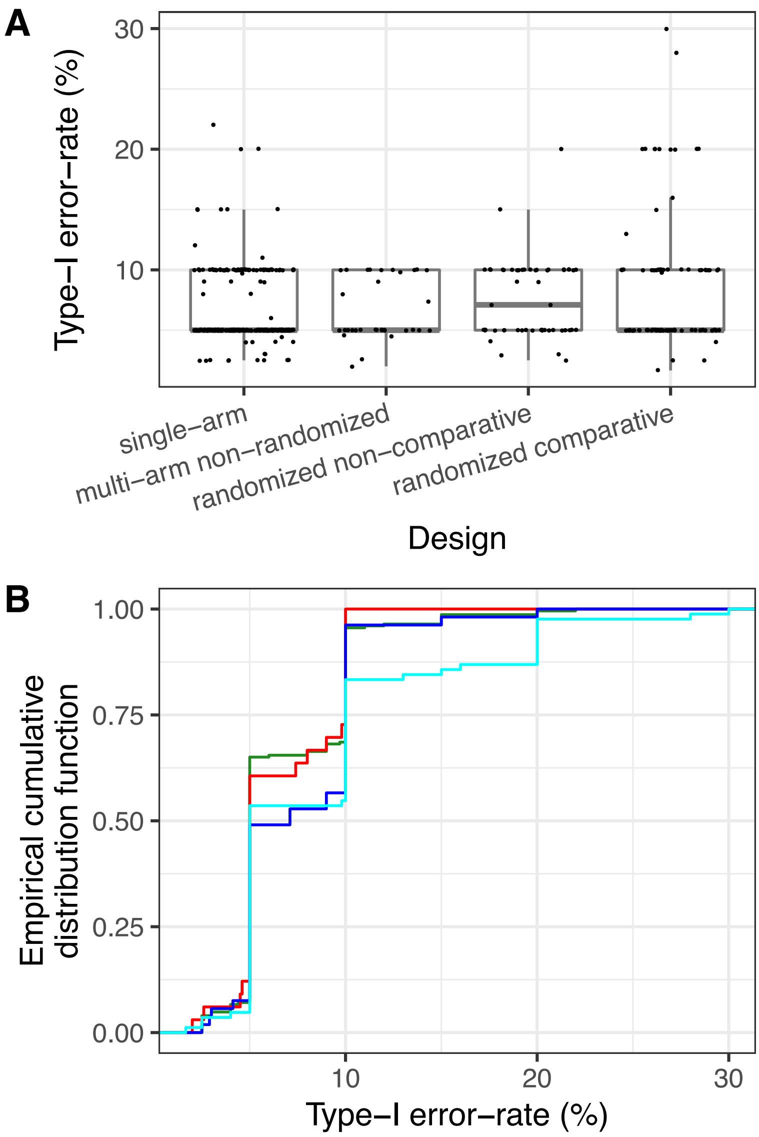

Supplementary Figure 1. Type-I error-rates of trials from Langrand-Escure et al. [80]. Depicts the distribution of the type-I error-rates utilized in the trials examined by Langrand-Escure et al. [80], via boxplots and empirical cumulative distribution functions. In each panel, the distributions are split according to the type of design used by the trial (e.g., single-arm). In panel B, the lines are colored as follows: single-arm – green, multi-arm non-randomised – red, randomized non-comparative – dark blue, randomized comparative – light blue.

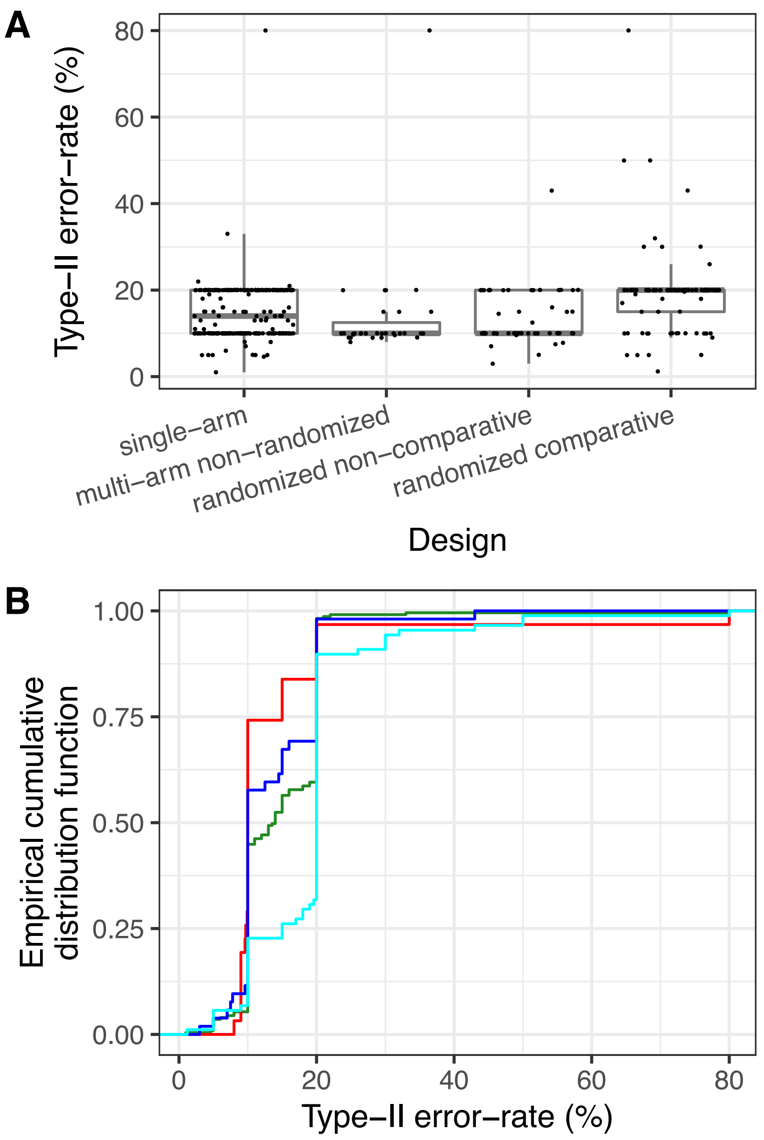

Supplementary Figure 2. Type-II error-rates of trials from Langrand-Escure et al. [80]. Depicts the distribution of the type-II error-rates utilized in the trials examined by Langrand-Escure et al. [80], via boxplots and empirical cumulative distribution functions. In each panel, the distributions are split according to the type of design used by the trial (e.g., single-arm). In panel B, the lines are colored as follows: single-arm – green, multi-arm non-randomised – red, randomized non-comparative – dark blue, randomized comparative – light blue.
